# Supplementary material for: Comparative multiomics analysis of cell physiological state after culture in a basket bioreactor
Source: Sci Rep. 2022 Nov 23;12:20161. doi: 10.1038/s41598-022-24687-4 (PMC9686226; doi:10.1038/s41598-022-24687-4)
Supplement: Supplementary file 1 — Supplementary Information 1. [file 41598_2022_24687_MOESM1_ESM.zip › raw data/Transcriptomic raw data/6.Enrichment/1.GO/TE1vsTC1/GSEA/TE1/NUCLEUS/NUCLEUS(GO_0005634).html]

Details for gene set NUCLEUS(GO:0005634)[GSEA]


|  || Dataset | readcount.sample |
| Phenotype | sample.cls |
| Upregulated in class | TE1 |
| GeneSet | NUCLEUS(GO:0005634) |
| Enrichment Score (ES) | 0.33138654 |
| Normalized Enrichment Score (NES) | 1.7732239 |
| Nominal p-value | 0.0 |
| FDR q-value | 0.21110101 |
| FWER p-Value | 0.494 |
Table: GSEA Results Summary

  

Fig 1: Enrichment plot: NUCLEUS(GO:0005634)
  
Profile of the Running ES Score & Positions of GeneSet Members on the Rank Ordered List

  

| PROBE | DESCRIPTION   (from dataset) | GENE SYMBOL | GENE\_TITLE | RANK IN GENE LIST | RANK METRIC SCORE | RUNNING ES | CORE ENRICHMENT || 1 | 103230021 | LOC103230021 |  |  | 66 | Infinity | -0.0030 | Yes |
| 2 | 119620737 | LOC119620737 |  |  | 181 | Infinity | -0.0082 | Yes |
| 3 | 119625749 | LOC119625749 |  |  | 193 | Infinity | -0.0087 | Yes |
| 4 | 103245400 | RBPJL |  |  | 369 | Infinity | -0.0167 | Yes |
| 5 | 103237494 | RAG2 |  |  | 388 | Infinity | -0.0175 | Yes |
| 6 | 119622594 | LOC119622594 |  |  | 479 | Infinity | -0.0216 | Yes |
| 7 | 103222013 | H1-4 |  |  | 615 | 12.838 | -0.0093 | Yes |
| 8 | 103219594 | RORB |  |  | 647 | 8.838 | 0.0021 | Yes |
| 9 | 103220985 | LOC103220985 |  |  | 654 | 8.330 | 0.0138 | Yes |
| 10 | 103229160 | ESR2 |  |  | 655 | 8.298 | 0.0258 | Yes |
| 11 | 103231918 | LOC103231918 |  |  | 656 | 8.256 | 0.0377 | Yes |
| 12 | 103219133 | NR4A3 |  |  | 674 | 7.430 | 0.0476 | Yes |
| 13 | 103236801 | LOC103236801 |  |  | 677 | 7.387 | 0.0582 | Yes |
| 14 | 103240625 | NR2E1 |  |  | 693 | 6.990 | 0.0675 | Yes |
| 15 | 103236678 | LOC103236678 |  |  | 700 | 6.610 | 0.0768 | Yes |
| 16 | 103238335 | NR4A1 |  |  | 821 | 4.959 | 0.0785 | Yes |
| 17 | 119619067 | LOC119619067 |  |  | 904 | 4.200 | 0.0808 | Yes |
| 18 | 103219305 | LOC103219305 |  |  | 938 | 3.944 | 0.0849 | Yes |
| 19 | 103246220 | PTTG2 |  |  | 974 | 3.744 | 0.0887 | Yes |
| 20 | 119625043 | LOC119625043 |  |  | 992 | 3.672 | 0.0933 | Yes |
| 21 | 103236228 | LOC103236228 |  |  | 1054 | 3.401 | 0.0954 | Yes |
| 22 | 103240497 | VGLL2 |  |  | 1116 | 3.270 | 0.0973 | Yes |
| 23 | 103232179 | CITED1 |  |  | 1169 | 3.112 | 0.0994 | Yes |
| 24 | 103236802 | TCEA1 |  |  | 1175 | 3.095 | 0.1036 | Yes |
| 25 | 103225266 | SESN2 |  |  | 1190 | 3.045 | 0.1074 | Yes |
| 26 | 103236990 | HNF4G |  |  | 1239 | 2.963 | 0.1095 | Yes |
| 27 | 103218035 | LOC103218035 |  |  | 1246 | 2.928 | 0.1134 | Yes |
| 28 | 103219739 | NFIL3 |  |  | 1255 | 2.899 | 0.1172 | Yes |
| 29 | 103228672 | LOC103228672 |  |  | 1264 | 2.879 | 0.1210 | Yes |
| 30 | 103247702 | LOC103247702 |  |  | 1273 | 2.862 | 0.1248 | Yes |
| 31 | 103238756 | NAP1L1 |  |  | 1283 | 2.845 | 0.1285 | Yes |
| 32 | 103240489 | LOC103240489 |  |  | 1288 | 2.834 | 0.1324 | Yes |
| 33 | 103245382 | NR2E3 |  |  | 1377 | 2.643 | 0.1321 | Yes |
| 34 | 103243618 | HOXB9 |  |  | 1388 | 2.621 | 0.1355 | Yes |
| 35 | 103237430 | MYC |  |  | 1413 | 2.559 | 0.1381 | Yes |
| 36 | 103216084 | DDX21 |  |  | 1507 | 2.442 | 0.1373 | Yes |
| 37 | 103218410 | CCND2 |  |  | 1569 | 2.397 | 0.1380 | Yes |
| 38 | 103228406 | CBLB |  |  | 1629 | 2.335 | 0.1387 | Yes |
| 39 | 103239188 | TBX3 |  |  | 1646 | 2.324 | 0.1413 | Yes |
| 40 | 103237174 | TSPYL5 |  |  | 1652 | 2.310 | 0.1444 | Yes |
| 41 | 103227841 | THOC7 |  |  | 1681 | 2.283 | 0.1464 | Yes |
| 42 | 119622140 | LOC119622140 |  |  | 1709 | 2.267 | 0.1484 | Yes |
| 43 | 103242850 | HSF5 |  |  | 1733 | 2.264 | 0.1506 | Yes |
| 44 | 103245731 | ELL3 |  |  | 1736 | 2.263 | 0.1538 | Yes |
| 45 | 103232650 | LOC103232650 |  |  | 1779 | 2.257 | 0.1551 | Yes |
| 46 | 103217123 | NR4A2 |  |  | 1792 | 2.244 | 0.1578 | Yes |
| 47 | 103244904 | CCNJL |  |  | 1810 | 2.220 | 0.1602 | Yes |
| 48 | 103216501 | SMNDC1 |  |  | 1842 | 2.194 | 0.1620 | Yes |
| 49 | 103235027 | TEAD2 |  |  | 1913 | 2.137 | 0.1619 | Yes |
| 50 | novel.575 | - |  |  | 1918 | 2.133 | 0.1648 | Yes |
| 51 | 103228546 | VGLL3 |  |  | 1937 | 2.120 | 0.1670 | Yes |
| 52 | 103232954 | LOC103232954 |  |  | 1977 | 2.097 | 0.1682 | Yes |
| 53 | 103222006 | H1-3 |  |  | 2009 | 2.080 | 0.1698 | Yes |
| 54 | 103222610 | TAF4B |  |  | 2022 | 2.072 | 0.1723 | Yes |
| 55 | 103237913 | ELF5 |  |  | 2044 | 2.053 | 0.1743 | Yes |
| 56 | 103221101 | ETV5 |  |  | 2049 | 2.049 | 0.1770 | Yes |
| 57 | 103248809 | ETS1 |  |  | 2071 | 2.035 | 0.1790 | Yes |
| 58 | 103237849 | SFMBT2 |  |  | 2084 | 2.026 | 0.1814 | Yes |
| 59 | 103224437 | LOC103224437 |  |  | 2185 | 1.993 | 0.1797 | Yes |
| 60 | 103241469 | MED12L |  |  | 2190 | 1.990 | 0.1824 | Yes |
| 61 | 119626531 | LOC119626531 |  |  | 2199 | 1.986 | 0.1849 | Yes |
| 62 | 103233022 | NUDT21 |  |  | 2321 | 1.926 | 0.1821 | Yes |
| 63 | 103227748 | MUSTN1 |  |  | 2360 | 1.908 | 0.1831 | Yes |
| 64 | 103232839 | LOC103232839 |  |  | 2393 | 1.899 | 0.1844 | Yes |
| 65 | 103226096 | HUS1 |  |  | 2431 | 1.885 | 0.1854 | Yes |
| 66 | 103224919 | ELL2 |  |  | 2437 | 1.882 | 0.1879 | Yes |
| 67 | 103244842 | CNOT8 |  |  | 2439 | 1.881 | 0.1906 | Yes |
| 68 | 103247026 | CSTF3 |  |  | 2460 | 1.872 | 0.1923 | Yes |
| 69 | 103241474 | SIAH2 |  |  | 2473 | 1.868 | 0.1945 | Yes |
| 70 | novel.578 | - |  |  | 2496 | 1.858 | 0.1962 | Yes |
| 71 | 103229604 | POLR3E |  |  | 2505 | 1.855 | 0.1985 | Yes |
| 72 | 103218585 | MAGOHB |  |  | 2522 | 1.848 | 0.2004 | Yes |
| 73 | 103243158 | NOL11 |  |  | 2566 | 1.834 | 0.2011 | Yes |
| 74 | 103214406 | RB1 |  |  | 2600 | 1.820 | 0.2022 | Yes |
| 75 | 103223309 | MAFF |  |  | 2636 | 1.808 | 0.2032 | Yes |
| 76 | 103221583 | CDKN1A |  |  | 2669 | 1.800 | 0.2043 | Yes |
| 77 | 103241328 | LOC103241328 |  |  | 2685 | 1.798 | 0.2062 | Yes |
| 78 | 103223057 | UTP15 |  |  | 2692 | 1.795 | 0.2085 | Yes |
| 79 | 119626588 | LOC119626588 |  |  | 2741 | 1.781 | 0.2089 | Yes |
| 80 | 103241791 | EOMES |  |  | 2753 | 1.776 | 0.2110 | Yes |
| 81 | 103244013 | TXNL4A |  |  | 2783 | 1.766 | 0.2122 | Yes |
| 82 | 103241764 | NR1D2 |  |  | 2952 | 1.718 | 0.2070 | Yes |
| 83 | 119622114 | LOC119622114 |  |  | 2964 | 1.715 | 0.2090 | Yes |
| 84 | 103218907 | HMGN1 |  |  | 2984 | 1.710 | 0.2105 | Yes |
| 85 | 103214463 | ESF1 |  |  | 3020 | 1.702 | 0.2114 | Yes |
| 86 | 119627658 | LOC119627658 |  |  | 3027 | 1.700 | 0.2136 | Yes |
| 87 | 103238651 | LEMD3 |  |  | 3035 | 1.700 | 0.2157 | Yes |
| 88 | 103221978 | HMGN4 |  |  | 3055 | 1.696 | 0.2173 | Yes |
| 89 | 103248629 | CBL |  |  | 3074 | 1.693 | 0.2189 | Yes |
| 90 | 103223292 | H1F0 |  |  | 3195 | 1.665 | 0.2158 | Yes |
| 91 | 103215388 | CNOT7 |  |  | 3237 | 1.658 | 0.2163 | Yes |
| 92 | 103241259 | ANAPC1 |  |  | 3280 | 1.650 | 0.2168 | Yes |
| 93 | 103220821 | NOL10 |  |  | 3298 | 1.646 | 0.2184 | Yes |
| 94 | 103236313 | SMARCA5 |  |  | 3299 | 1.646 | 0.2208 | Yes |
| 95 | 103241991 | LOC103241991 |  |  | 3319 | 1.641 | 0.2222 | Yes |
| 96 | 103243522 | NR1D1 |  |  | 3324 | 1.640 | 0.2244 | Yes |
| 97 | 103240386 | L3MBTL3 |  |  | 3365 | 1.634 | 0.2250 | Yes |
| 98 | 103214372 | GTF2F2 |  |  | 3426 | 1.622 | 0.2246 | Yes |
| 99 | 103222315 | HUS1B |  |  | 3444 | 1.620 | 0.2261 | Yes |
| 100 | 103230150 | UTP25 |  |  | 3475 | 1.614 | 0.2271 | Yes |
| 101 | 103232533 | SMARCA1 |  |  | 3518 | 1.606 | 0.2275 | Yes |
| 102 | 103240001 | TEAD1 |  |  | 3556 | 1.599 | 0.2281 | Yes |
| 103 | 103245539 | GTF2A2 |  |  | 3567 | 1.597 | 0.2299 | Yes |
| 104 | 103215581 | LOC103215581 |  |  | 3610 | 1.590 | 0.2303 | Yes |
| 105 | 103242997 | LOC103242997 |  |  | 3716 | 1.571 | 0.2277 | Yes |
| 106 | 103222405 | MBD2 |  |  | 3730 | 1.569 | 0.2294 | Yes |
| 107 | 103228069 | MBD4 |  |  | 3745 | 1.567 | 0.2310 | Yes |
| 108 | 103242258 | MED31 |  |  | 3746 | 1.567 | 0.2333 | Yes |
| 109 | 103243195 | DDX5 |  |  | 3798 | 1.556 | 0.2332 | Yes |
| 110 | 103222342 | CENPK |  |  | 3865 | 1.546 | 0.2324 | Yes |
| 111 | 103229118 | MNAT1 |  |  | 3869 | 1.546 | 0.2345 | Yes |
| 112 | 103230666 | TADA1 |  |  | 3875 | 1.544 | 0.2365 | Yes |
| 113 | 103216315 | CCNJ |  |  | 3927 | 1.537 | 0.2364 | Yes |
| 114 | 103217801 | GABPA |  |  | 3940 | 1.535 | 0.2380 | Yes |
| 115 | 103234861 | ERCC2 |  |  | 3942 | 1.535 | 0.2402 | Yes |
| 116 | 103240454 | HSF2 |  |  | 3982 | 1.529 | 0.2406 | Yes |
| 117 | 103240502 | KPNA5 |  |  | 4156 | 1.508 | 0.2349 | Yes |
| 118 | 103241775 | TOP2B |  |  | 4157 | 1.508 | 0.2371 | Yes |
| 119 | 103245452 | TIPIN |  |  | 4161 | 1.508 | 0.2391 | Yes |
| 120 | 103225096 | MEAF6 |  |  | 4171 | 1.506 | 0.2409 | Yes |
| 121 | 103214447 | RNASEH2B |  |  | 4238 | 1.495 | 0.2400 | Yes |
| 122 | 103228108 | MCM2 |  |  | 4280 | 1.489 | 0.2403 | Yes |
| 123 | 103246138 | NFXL1 |  |  | 4301 | 1.485 | 0.2415 | Yes |
| 124 | 103221494 | CCNO |  |  | 4319 | 1.483 | 0.2429 | Yes |
| 125 | 103245531 | ICE2 |  |  | 4424 | 1.470 | 0.2402 | Yes |
| 126 | 103232283 | ZNF711 |  |  | 4455 | 1.466 | 0.2410 | Yes |
| 127 | 103238386 | RARG |  |  | 4464 | 1.465 | 0.2427 | Yes |
| 128 | 103240279 | CITED2 |  |  | 4511 | 1.458 | 0.2427 | Yes |
| 129 | 103216455 | TAF5 |  |  | 4542 | 1.454 | 0.2434 | Yes |
| 130 | 103244720 | NR3C1 |  |  | 4562 | 1.452 | 0.2446 | Yes |
| 131 | 103247974 | NUP98 |  |  | 4638 | 1.442 | 0.2433 | Yes |
| 132 | 103233302 | CBFB |  |  | 4649 | 1.441 | 0.2449 | Yes |
| 133 | 103226343 | CBX3 |  |  | 4697 | 1.436 | 0.2448 | Yes |
| 134 | 103232443 | SIAH1 |  |  | 4733 | 1.432 | 0.2453 | Yes |
| 135 | 103244690 | TAF7 |  |  | 4742 | 1.431 | 0.2470 | Yes |
| 136 | 103242815 | MED13 |  |  | 4822 | 1.420 | 0.2454 | Yes |
| 137 | 103239198 | MED13L |  |  | 4848 | 1.417 | 0.2463 | Yes |
| 138 | 103214281 | CCNA1 |  |  | 4889 | 1.412 | 0.2465 | Yes |
| 139 | 103222141 | NOL7 |  |  | 4916 | 1.410 | 0.2474 | Yes |
| 140 | 103238886 | NR2C1 |  |  | 4956 | 1.407 | 0.2476 | Yes |
| 141 | 103236303 | ZNF330 |  |  | 4969 | 1.405 | 0.2491 | Yes |
| 142 | 103228027 | PPARG |  |  | 4981 | 1.403 | 0.2506 | Yes |
| 143 | 103231818 | MED14 |  |  | 4996 | 1.401 | 0.2520 | Yes |
| 144 | 103237327 | RAD21 |  |  | 5038 | 1.397 | 0.2521 | Yes |
| 145 | 119619406 | LOC119619406 |  |  | 5039 | 1.397 | 0.2541 | Yes |
| 146 | 103224082 | XRCC4 |  |  | 5055 | 1.395 | 0.2555 | Yes |
| 147 | 103221505 | MED20 |  |  | 5106 | 1.390 | 0.2552 | Yes |
| 148 | 119625232 | GTF2H5 |  |  | 5113 | 1.390 | 0.2569 | Yes |
| 149 | 103242681 | COPRS |  |  | 5155 | 1.385 | 0.2570 | Yes |
| 150 | 103228184 | KPNA1 |  |  | 5210 | 1.378 | 0.2566 | Yes |
| 151 | 103236758 | PRKDC |  |  | 5225 | 1.376 | 0.2579 | Yes |
| 152 | 103248819 | FLI1 |  |  | 5232 | 1.376 | 0.2596 | Yes |
| 153 | 103225790 | SSU72 |  |  | 5287 | 1.370 | 0.2591 | Yes |
| 154 | 103219512 | RFX3 |  |  | 5305 | 1.368 | 0.2603 | Yes |
| 155 | 103242814 | INTS2 |  |  | 5353 | 1.363 | 0.2601 | Yes |
| 156 | 103216456 | PDCD11 |  |  | 5386 | 1.360 | 0.2606 | Yes |
| 157 | 119620933 | LOC119620933 |  |  | 5415 | 1.358 | 0.2613 | Yes |
| 158 | 103233191 | TXNL4B |  |  | 5418 | 1.358 | 0.2632 | Yes |
| 159 | 103229271 | MED6 |  |  | 5428 | 1.357 | 0.2647 | Yes |
| 160 | 103228057 | NR2C2 |  |  | 5509 | 1.348 | 0.2630 | Yes |
| 161 | 103220005 | GCFC2 |  |  | 5538 | 1.344 | 0.2636 | Yes |
| 162 | 103246453 | LOC103246453 |  |  | 5668 | 1.333 | 0.2597 | Yes |
| 163 | 103243219 | FTSJ3 |  |  | 5689 | 1.332 | 0.2607 | Yes |
| 164 | 103235959 | NAP1L5 |  |  | 5724 | 1.329 | 0.2610 | Yes |
| 165 | 103221740 | GPBP1 |  |  | 5733 | 1.328 | 0.2626 | Yes |
| 166 | 103223491 | LOC103223491 |  |  | 5812 | 1.320 | 0.2609 | Yes |
| 167 | 103248320 | PGR |  |  | 5838 | 1.319 | 0.2617 | Yes |
| 168 | 103217623 | ORC2 |  |  | 5844 | 1.318 | 0.2633 | Yes |
| 169 | 103220076 | MPHOSPH10 |  |  | 5847 | 1.318 | 0.2652 | Yes |
| 170 | 103217174 | TBR1 |  |  | 5877 | 1.313 | 0.2657 | Yes |
| 171 | 103231674 | SCML2 |  |  | 5922 | 1.309 | 0.2656 | Yes |
| 172 | 103219181 | POLR1E |  |  | 5929 | 1.308 | 0.2672 | Yes |
| 173 | 103230089 | ESRRG |  |  | 5947 | 1.306 | 0.2683 | Yes |
| 174 | 103230699 | PBX1 |  |  | 5963 | 1.305 | 0.2695 | Yes |
| 175 | 103222020 | H1-2 |  |  | 5966 | 1.305 | 0.2713 | Yes |
| 176 | 103223006 | HIRA |  |  | 6014 | 1.301 | 0.2710 | Yes |
| 177 | 103243867 | MED1 |  |  | 6025 | 1.300 | 0.2724 | Yes |
| 178 | 103242909 | MBTD1 |  |  | 6034 | 1.298 | 0.2739 | Yes |
| 179 | 103225702 | CHD5 |  |  | 6115 | 1.292 | 0.2721 | Yes |
| 180 | 103216085 | DDX50 |  |  | 6128 | 1.291 | 0.2735 | Yes |
| 181 | 103216948 | PAXBP1 |  |  | 6151 | 1.288 | 0.2743 | Yes |
| 182 | 103241767 | THRB |  |  | 6169 | 1.286 | 0.2754 | Yes |
| 183 | 103227400 | NUP205 |  |  | 6216 | 1.282 | 0.2751 | Yes |
| 184 | 103214430 | KPNA3 |  |  | 6252 | 1.278 | 0.2754 | Yes |
| 185 | 103247740 | LOC103247740 |  |  | 6405 | 1.271 | 0.2702 | Yes |
| 186 | 103223028 | SMARCB1 |  |  | 6407 | 1.270 | 0.2720 | Yes |
| 187 | 103248447 | CUNH11orf1 |  |  | 6415 | 1.270 | 0.2735 | Yes |
| 188 | 103246782 | BUD31 |  |  | 6434 | 1.268 | 0.2745 | Yes |
| 189 | 103248594 | KMT2A |  |  | 6458 | 1.266 | 0.2753 | Yes |
| 190 | 103216878 | IWS1 |  |  | 6491 | 1.264 | 0.2757 | Yes |
| 191 | 103238554 | NAB2 |  |  | 6496 | 1.263 | 0.2773 | Yes |
| 192 | 103245110 | MAML1 |  |  | 6508 | 1.262 | 0.2786 | Yes |
| 193 | 103225390 | ELOA |  |  | 6514 | 1.261 | 0.2802 | Yes |
| 194 | 103225204 | KPNA6 |  |  | 6560 | 1.257 | 0.2800 | Yes |
| 195 | 103224460 | ZNF326 |  |  | 6561 | 1.257 | 0.2818 | Yes |
| 196 | 103215115 | RAD1 |  |  | 6595 | 1.253 | 0.2821 | Yes |
| 197 | 103220306 | STON1 |  |  | 6604 | 1.252 | 0.2835 | Yes |
| 198 | 103239544 | EHMT1 |  |  | 6605 | 1.252 | 0.2853 | Yes |
| 199 | 103238534 | BAZ2A |  |  | 6608 | 1.252 | 0.2870 | Yes |
| 200 | 103233854 | NXF1 |  |  | 6631 | 1.249 | 0.2878 | Yes |
| 201 | 103214287 | SUPT20H |  |  | 6634 | 1.249 | 0.2895 | Yes |
| 202 | 119620241 | LOC119620241 |  |  | 6671 | 1.245 | 0.2897 | Yes |
| 203 | 103216324 | DNTT |  |  | 6716 | 1.242 | 0.2895 | Yes |
| 204 | 103227751 | SFMBT1 |  |  | 6731 | 1.241 | 0.2906 | Yes |
| 205 | 103224681 | NR2F1 |  |  | 6748 | 1.240 | 0.2917 | Yes |
| 206 | 103239334 | GTF2H3 |  |  | 6791 | 1.237 | 0.2915 | Yes |
| 207 | 103239559 | NELFB |  |  | 6807 | 1.236 | 0.2926 | Yes |
| 208 | 103222822 | LOC103222822 |  |  | 6812 | 1.235 | 0.2942 | Yes |
| 209 | 103241693 | NAP1L4 |  |  | 6835 | 1.234 | 0.2950 | Yes |
| 210 | 103225446 | HP1BP3 |  |  | 6847 | 1.233 | 0.2963 | Yes |
| 211 | 103243455 | NELFCD |  |  | 6852 | 1.233 | 0.2979 | Yes |
| 212 | 103218838 | ETS2 |  |  | 6901 | 1.229 | 0.2975 | Yes |
| 213 | 103222804 | CCNB1 |  |  | 6913 | 1.228 | 0.2987 | Yes |
| 214 | 103248266 | CWC15 |  |  | 6927 | 1.228 | 0.2999 | Yes |
| 215 | 103240483 | ASF1A |  |  | 6931 | 1.227 | 0.3015 | Yes |
| 216 | 103240031 | TBX18 |  |  | 6981 | 1.223 | 0.3010 | Yes |
| 217 | 103248938 | ZW10 |  |  | 6995 | 1.222 | 0.3022 | Yes |
| 218 | 103229866 | RBBP6 |  |  | 7024 | 1.219 | 0.3027 | Yes |
| 219 | 103248243 | MED17 |  |  | 7047 | 1.217 | 0.3034 | Yes |
| 220 | 103237144 | LOC103237144 |  |  | 7078 | 1.215 | 0.3038 | Yes |
| 221 | 119621789 | LOC119621789 |  |  | 7181 | 1.206 | 0.3009 | Yes |
| 222 | 103233159 | SF3B3 |  |  | 7193 | 1.205 | 0.3021 | Yes |
| 223 | 103225612 | EXOSC10 |  |  | 7203 | 1.205 | 0.3034 | Yes |
| 224 | 103217393 | NFE2L2 |  |  | 7260 | 1.200 | 0.3026 | Yes |
| 225 | 103218658 | SIM2 |  |  | 7269 | 1.199 | 0.3040 | Yes |
| 226 | 103242779 | HNF1B |  |  | 7338 | 1.194 | 0.3026 | Yes |
| 227 | 103248238 | CUNH11orf54 |  |  | 7365 | 1.193 | 0.3031 | Yes |
| 228 | 103224965 | MED8 |  |  | 7374 | 1.192 | 0.3045 | Yes |
| 229 | 103228076 | H1FX |  |  | 7401 | 1.190 | 0.3050 | Yes |
| 230 | 103224714 | NFIA |  |  | 7428 | 1.188 | 0.3055 | Yes |
| 231 | 103226889 | ZC3HC1 |  |  | 7429 | 1.187 | 0.3072 | Yes |
| 232 | 103217895 | NHEJ1 |  |  | 7438 | 1.186 | 0.3086 | Yes |
| 233 | 103236289 | MAML3 |  |  | 7443 | 1.186 | 0.3101 | Yes |
| 234 | 103247451 | ZFX |  |  | 7451 | 1.185 | 0.3115 | Yes |
| 235 | 103235327 | MED19 |  |  | 7454 | 1.185 | 0.3131 | Yes |
| 236 | 103214895 | POLR3F |  |  | 7489 | 1.182 | 0.3133 | Yes |
| 237 | 103245800 | MGA |  |  | 7617 | 1.173 | 0.3091 | Yes |
| 238 | 103245565 | POLR2M |  |  | 7621 | 1.172 | 0.3107 | Yes |
| 239 | 103244615 | CDC23 |  |  | 7627 | 1.171 | 0.3122 | Yes |
| 240 | 103229533 | TDP1 |  |  | 7635 | 1.171 | 0.3135 | Yes |
| 241 | 103234375 | RFX1 |  |  | 7639 | 1.170 | 0.3151 | Yes |
| 242 | 103226688 | ORC5 |  |  | 7642 | 1.170 | 0.3167 | Yes |
| 243 | 103239865 | PBX3 |  |  | 7651 | 1.169 | 0.3180 | Yes |
| 244 | 103244688 | NCOA3 |  |  | 7687 | 1.167 | 0.3181 | Yes |
| 245 | 103225252 | TAF12 |  |  | 7699 | 1.166 | 0.3192 | Yes |
| 246 | 103227268 | KMT2C |  |  | 7701 | 1.166 | 0.3209 | Yes |
| 247 | 103234925 | LOC103234925 |  |  | 7748 | 1.162 | 0.3204 | Yes |
| 248 | 103243599 | CBX1 |  |  | 7749 | 1.162 | 0.3221 | Yes |
| 249 | 103226346 | NFE2L3 |  |  | 7784 | 1.158 | 0.3222 | Yes |
| 250 | 103232914 | RBL2 |  |  | 7847 | 1.155 | 0.3211 | Yes |
| 251 | 103243741 | MAFG |  |  | 7853 | 1.154 | 0.3225 | Yes |
| 252 | 103245495 | TRIP4 |  |  | 7855 | 1.154 | 0.3241 | Yes |
| 253 | 103240606 | SESN1 |  |  | 7887 | 1.151 | 0.3244 | Yes |
| 254 | 103215753 | BMS1 |  |  | 7938 | 1.148 | 0.3237 | Yes |
| 255 | 103230728 | TAF5L |  |  | 7980 | 1.145 | 0.3235 | Yes |
| 256 | 103222722 | L3MBTL4 |  |  | 7983 | 1.144 | 0.3251 | Yes |
| 257 | 103229400 | SNW1 |  |  | 7993 | 1.144 | 0.3263 | Yes |
| 258 | 103243423 | KAT2A |  |  | 8001 | 1.143 | 0.3276 | Yes |
| 259 | 103225226 | LOC103225226 |  |  | 8014 | 1.142 | 0.3287 | Yes |
| 260 | 103243153 | KPNA2 |  |  | 8015 | 1.142 | 0.3304 | Yes |
| 261 | 103218996 | CDC26 |  |  | 8044 | 1.139 | 0.3307 | Yes |
| 262 | 103238683 | NUP107 |  |  | 8106 | 1.134 | 0.3296 | Yes |
| 263 | 103214423 | SETDB2 |  |  | 8202 | 1.132 | 0.3269 | Yes |
| 264 | 103240678 | SIM1 |  |  | 8300 | 1.129 | 0.3241 | Yes |
| 265 | 103224108 | LOC103224108 |  |  | 8345 | 1.129 | 0.3237 | Yes |
| 266 | 103245448 | SNAPC5 |  |  | 8362 | 1.129 | 0.3246 | Yes |
| 267 | 103242963 | KAT7 |  |  | 8379 | 1.127 | 0.3255 | Yes |
| 268 | 103238486 | LOC103238486 |  |  | 8421 | 1.125 | 0.3252 | Yes |
| 269 | 103225671 | CAMTA1 |  |  | 8449 | 1.122 | 0.3256 | Yes |
| 270 | 119626586 | LOC119626586 |  |  | 8481 | 1.120 | 0.3258 | Yes |
| 271 | 103215450 | POLR3D |  |  | 8526 | 1.116 | 0.3254 | Yes |
| 272 | 103237623 | CPSF1 |  |  | 8551 | 1.114 | 0.3259 | Yes |
| 273 | 103241393 | KPNA4 |  |  | 8569 | 1.113 | 0.3267 | Yes |
| 274 | 103239886 | NR6A1 |  |  | 8603 | 1.111 | 0.3268 | Yes |
| 275 | 103226491 | RPA3 |  |  | 8661 | 1.107 | 0.3258 | Yes |
| 276 | 103223468 | NUP50 |  |  | 8667 | 1.106 | 0.3272 | Yes |
| 277 | 103237617 | HSF1 |  |  | 8670 | 1.106 | 0.3287 | Yes |
| 278 | 103214401 | MED4 |  |  | 8713 | 1.103 | 0.3283 | Yes |
| 279 | 103241270 | POLR1B |  |  | 8739 | 1.102 | 0.3288 | Yes |
| 280 | 103240206 | SHPRH |  |  | 8771 | 1.099 | 0.3289 | Yes |
| 281 | 103248239 | TAF1D |  |  | 8796 | 1.098 | 0.3294 | Yes |
| 282 | 103238711 | CNOT2 |  |  | 8804 | 1.097 | 0.3307 | Yes |
| 283 | 103234005 | GADD45GIP1 |  |  | 8867 | 1.094 | 0.3294 | Yes |
| 284 | 103232817 | MECP2 |  |  | 8885 | 1.093 | 0.3302 | Yes |
| 285 | 103237926 | SUV39H2 |  |  | 8920 | 1.091 | 0.3302 | Yes |
| 286 | 103226799 | LOC103226799 |  |  | 9050 | 1.082 | 0.3259 | Yes |
| 287 | 103228706 | NRL |  |  | 9125 | 1.077 | 0.3241 | Yes |
| 288 | 103236905 | RRS1 |  |  | 9163 | 1.075 | 0.3239 | Yes |
| 289 | 103239271 | HNF1A |  |  | 9234 | 1.070 | 0.3223 | Yes |
| 290 | 103234463 | DDB1 |  |  | 9311 | 1.065 | 0.3203 | Yes |
| 291 | 103215556 | HMBOX1 |  |  | 9348 | 1.063 | 0.3202 | Yes |
| 292 | 103242794 | AATF |  |  | 9396 | 1.060 | 0.3196 | Yes |
| 293 | 103232318 | NAP1L3 |  |  | 9413 | 1.059 | 0.3204 | Yes |
| 294 | 103225016 | SCMH1 |  |  | 9457 | 1.057 | 0.3199 | Yes |
| 295 | 103226830 | POT1 |  |  | 9506 | 1.053 | 0.3193 | Yes |
| 296 | 103221675 | RXRB |  |  | 9515 | 1.053 | 0.3204 | Yes |
| 297 | 103214967 | MED10 |  |  | 9599 | 1.048 | 0.3181 | Yes |
| 298 | 103224113 | TBX15 |  |  | 9601 | 1.048 | 0.3196 | Yes |
| 299 | 119627346 | LOC119627346 |  |  | 9650 | 1.044 | 0.3189 | Yes |
| 300 | 119627856 | LOC119627856 |  |  | 9652 | 1.044 | 0.3204 | Yes |
| 301 | 103229444 | GTF2A1 |  |  | 9653 | 1.044 | 0.3219 | Yes |
| 302 | 103240819 | LOC103240819 |  |  | 9666 | 1.043 | 0.3228 | Yes |
| 303 | 103224792 | MAGOH |  |  | 9731 | 1.039 | 0.3214 | Yes |
| 304 | 103223371 | EP300 |  |  | 9745 | 1.038 | 0.3223 | Yes |
| 305 | 103236358 | NR3C2 |  |  | 9773 | 1.037 | 0.3225 | Yes |
| 306 | 103246721 | POP7 |  |  | 9780 | 1.036 | 0.3238 | Yes |
| 307 | 103217455 | BACH1 |  |  | 9783 | 1.036 | 0.3252 | Yes |
| 308 | 103223170 | PES1 |  |  | 9881 | 1.030 | 0.3222 | Yes |
| 309 | 103242235 | MIS12 |  |  | 9891 | 1.029 | 0.3233 | Yes |
| 310 | 103245617 | LEO1 |  |  | 9893 | 1.029 | 0.3247 | Yes |
| 311 | 119621747 | LOC119621747 |  |  | 9898 | 1.029 | 0.3260 | Yes |
| 312 | 103237901 | MCM10 |  |  | 9921 | 1.027 | 0.3265 | Yes |
| 313 | 103234072 | AKAP8 |  |  | 9962 | 1.023 | 0.3261 | Yes |
| 314 | 103235078 | NR1H2 |  |  | 9978 | 1.022 | 0.3269 | Yes |
| 315 | 103241748 | KAT2B |  |  | 9980 | 1.022 | 0.3284 | Yes |
| 316 | 103248038 | POLD3 |  |  | 9995 | 1.021 | 0.3292 | Yes |
| 317 | 103218388 | RHNO1 |  |  | 10013 | 1.019 | 0.3299 | Yes |
| 318 | 103233046 | NUP93 |  |  | 10031 | 1.018 | 0.3306 | Yes |
| 319 | 103248973 | PRPF6 |  |  | 10055 | 1.015 | 0.3310 | Yes |
| 320 | 103218647 | CDKN1B |  |  | 10079 | 1.014 | 0.3314 | Yes |
| 321 | 103235245 | CNOT3 |  |  | 10162 | 1.008 | 0.3291 | No |
| 322 | 103248255 | MRE11 |  |  | 10272 | 1.006 | 0.3256 | No |
| 323 | 103231770 | LOC103231770 |  |  | 10273 | 1.006 | 0.3270 | No |
| 324 | 103242922 | LUC7L3 |  |  | 10321 | 1.002 | 0.3263 | No |
| 325 | 103219284 | LOC103219284 |  |  | 10369 | 0.999 | 0.3256 | No |
| 326 | 103236261 | LOC103236261 |  |  | 10505 | 0.990 | 0.3208 | No |
| 327 | 103242330 | CHD3 |  |  | 10533 | 0.989 | 0.3210 | No |
| 328 | 103228729 | PARN |  |  | 10578 | 0.985 | 0.3204 | No |
| 329 | 103220176 | OTX1 |  |  | 10613 | 0.983 | 0.3203 | No |
| 330 | 103222426 | MBD1 |  |  | 10618 | 0.983 | 0.3215 | No |
| 331 | 103215689 | POLB |  |  | 10637 | 0.982 | 0.3221 | No |
| 332 | 103228714 | REC8 |  |  | 10733 | 0.974 | 0.3192 | No |
| 333 | 103243521 | THRA |  |  | 10746 | 0.973 | 0.3200 | No |
| 334 | 103232731 | EOLA2 |  |  | 10759 | 0.972 | 0.3209 | No |
| 335 | 103234295 | LOC103234295 |  |  | 10779 | 0.971 | 0.3214 | No |
| 336 | 103221737 | EHMT2 |  |  | 10813 | 0.968 | 0.3213 | No |
| 337 | 103249020 | TAF4 |  |  | 10825 | 0.968 | 0.3222 | No |
| 338 | 103243030 | GATA5 |  |  | 10864 | 0.965 | 0.3218 | No |
| 339 | 103242545 | RPA1 |  |  | 10865 | 0.965 | 0.3232 | No |
| 340 | 103243295 | HEXIM1 |  |  | 10924 | 0.962 | 0.3220 | No |
| 341 | 103232184 | ORC6 |  |  | 10962 | 0.959 | 0.3217 | No |
| 342 | 103238253 | KMT2D |  |  | 10995 | 0.958 | 0.3216 | No |
| 343 | 103221615 | PPARD |  |  | 10998 | 0.957 | 0.3229 | No |
| 344 | 103233027 | MEN1 |  |  | 11000 | 0.957 | 0.3242 | No |
| 345 | 103246756 | TAF6 |  |  | 11010 | 0.956 | 0.3252 | No |
| 346 | 103233917 | SMARCA4 |  |  | 11094 | 0.950 | 0.3227 | No |
| 347 | 103246728 | SAP25 |  |  | 11100 | 0.950 | 0.3239 | No |
| 348 | 103221450 | LOC103221450 |  |  | 11102 | 0.950 | 0.3252 | No |
| 349 | 103229907 | LIN52 |  |  | 11104 | 0.950 | 0.3265 | No |
| 350 | 103240111 | BACH2 |  |  | 11121 | 0.949 | 0.3272 | No |
| 351 | 103224630 | SF3B2 |  |  | 11130 | 0.948 | 0.3282 | No |
| 352 | 119623037 | LOC119623037 |  |  | 11190 | 0.945 | 0.3268 | No |
| 353 | 103221609 | TEAD3 |  |  | 11332 | 0.936 | 0.3217 | No |
| 354 | 103247223 | SETDB1 |  |  | 11365 | 0.934 | 0.3216 | No |
| 355 | 103231188 | BLM |  |  | 11372 | 0.934 | 0.3227 | No |
| 356 | 103232264 | HMGN5 |  |  | 11388 | 0.933 | 0.3233 | No |
| 357 | 103229121 | TRMT5 |  |  | 11450 | 0.929 | 0.3219 | No |
| 358 | 103243722 | FAAP100 |  |  | 11580 | 0.921 | 0.3173 | No |
| 359 | 103244860 | MED7 |  |  | 11585 | 0.921 | 0.3185 | No |
| 360 | 103245548 | CCNB2 |  |  | 11587 | 0.920 | 0.3197 | No |
| 361 | 103245526 | RORA |  |  | 11632 | 0.917 | 0.3190 | No |
| 362 | 103215728 | LOC103215728 |  |  | 11644 | 0.917 | 0.3199 | No |
| 363 | 103246259 | RBPJ |  |  | 11649 | 0.916 | 0.3210 | No |
| 364 | 103239455 | POLE |  |  | 11657 | 0.916 | 0.3220 | No |
| 365 | 103225269 | LUC7L |  |  | 11743 | 0.910 | 0.3194 | No |
| 366 | 103239161 | DDX54 |  |  | 11754 | 0.909 | 0.3203 | No |
| 367 | 103226328 | HOXA9 |  |  | 11778 | 0.908 | 0.3205 | No |
| 368 | 103222970 | MED15 |  |  | 11789 | 0.907 | 0.3214 | No |
| 369 | 103247439 | FAM50A |  |  | 11810 | 0.906 | 0.3218 | No |
| 370 | 103240070 | ORC3 |  |  | 11813 | 0.906 | 0.3230 | No |
| 371 | 103225722 | DFFB |  |  | 11822 | 0.905 | 0.3239 | No |
| 372 | 103234670 | PAF1 |  |  | 11860 | 0.902 | 0.3235 | No |
| 373 | 103239694 | MED22 |  |  | 11890 | 0.900 | 0.3235 | No |
| 374 | 103234668 | MED29 |  |  | 11948 | 0.897 | 0.3222 | No |
| 375 | 103245960 | LOC103245960 |  |  | 11985 | 0.895 | 0.3218 | No |
| 376 | 103243593 | NFE2L1 |  |  | 12032 | 0.892 | 0.3210 | No |
| 377 | 103243360 | ETV4 |  |  | 12036 | 0.892 | 0.3222 | No |
| 378 | 103221710 | PBX2 |  |  | 12045 | 0.891 | 0.3231 | No |
| 379 | 103243515 | RARA |  |  | 12086 | 0.889 | 0.3225 | No |
| 380 | 103217524 | NAB1 |  |  | 12113 | 0.887 | 0.3226 | No |
| 381 | 103235482 | CCNE1 |  |  | 12129 | 0.886 | 0.3232 | No |
| 382 | 103236201 | CCNA2 |  |  | 12131 | 0.886 | 0.3244 | No |
| 383 | 103243514 | TOP2A |  |  | 12133 | 0.885 | 0.3257 | No |
| 384 | 103241749 | SGO1 |  |  | 12197 | 0.882 | 0.3241 | No |
| 385 | 103226231 | TBX20 |  |  | 12225 | 0.880 | 0.3241 | No |
| 386 | 103232009 | TSPYL2 |  |  | 12281 | 0.877 | 0.3228 | No |
| 387 | 103234125 | NR2F6 |  |  | 12312 | 0.875 | 0.3227 | No |
| 388 | 103238431 | CBX5 |  |  | 12321 | 0.874 | 0.3236 | No |
| 389 | 103219433 | LOC103219433 |  |  | 12478 | 0.864 | 0.3177 | No |
| 390 | 103215919 | RAD21L1 |  |  | 12624 | 0.854 | 0.3123 | No |
| 391 | 103218457 | CHD4 |  |  | 12643 | 0.853 | 0.3127 | No |
| 392 | 119621569 | LOC119621569 |  |  | 12662 | 0.852 | 0.3131 | No |
| 393 | 103234071 | AKAP8L |  |  | 12796 | 0.845 | 0.3083 | No |
| 394 | 103218836 | LOC103218836 |  |  | 12802 | 0.844 | 0.3093 | No |
| 395 | 103220667 | NCOA1 |  |  | 12829 | 0.843 | 0.3093 | No |
| 396 | 103241314 | TAF10 |  |  | 12837 | 0.842 | 0.3102 | No |
| 397 | 103237915 | PRPF18 |  |  | 12881 | 0.840 | 0.3094 | No |
| 398 | 103232147 | MED12 |  |  | 12883 | 0.840 | 0.3106 | No |
| 399 | 103224623 | LOC103224623 |  |  | 12892 | 0.840 | 0.3114 | No |
| 400 | 103225956 | DHDDS |  |  | 12946 | 0.836 | 0.3102 | No |
| 401 | 103233869 | TAF6L |  |  | 12968 | 0.835 | 0.3105 | No |
| 402 | 103234378 | ASF1B |  |  | 12972 | 0.834 | 0.3115 | No |
| 403 | 103218036 | SP100 |  |  | 12987 | 0.834 | 0.3121 | No |
| 404 | 103245792 | L3MBTL1 |  |  | 12990 | 0.833 | 0.3132 | No |
| 405 | 103248916 | LOC103248916 |  |  | 13017 | 0.832 | 0.3132 | No |
| 406 | 103242218 | CAMTA2 |  |  | 13064 | 0.830 | 0.3123 | No |
| 407 | 103239966 | HMGN3 |  |  | 13079 | 0.829 | 0.3129 | No |
| 408 | 103224942 | DMAP1 |  |  | 13117 | 0.826 | 0.3124 | No |
| 409 | 103243018 | TEN1 |  |  | 13123 | 0.825 | 0.3133 | No |
| 410 | 103221648 | LEMD2 |  |  | 13190 | 0.821 | 0.3115 | No |
| 411 | 103234531 | KMT2B |  |  | 13233 | 0.818 | 0.3107 | No |
| 412 | 103247532 | CENPB |  |  | 13389 | 0.808 | 0.3048 | No |
| 413 | 103231980 | CCNB3 |  |  | 13405 | 0.807 | 0.3053 | No |
| 414 | 103223375 | L3MBTL2 |  |  | 13434 | 0.804 | 0.3052 | No |
| 415 | 103244908 | PTTG1 |  |  | 13458 | 0.803 | 0.3053 | No |
| 416 | 103226459 | ETV1 |  |  | 13480 | 0.802 | 0.3055 | No |
| 417 | 103218636 | ETV6 |  |  | 13502 | 0.801 | 0.3057 | No |
| 418 | 103234111 | MED26 |  |  | 13633 | 0.793 | 0.3009 | No |
| 419 | 103243735 | ANAPC11 |  |  | 13752 | 0.785 | 0.2966 | No |
| 420 | 103248163 | LOC103248163 |  |  | 13832 | 0.779 | 0.2941 | No |
| 421 | 119623427 | LOC119623427 |  |  | 13849 | 0.778 | 0.2945 | No |
| 422 | 103221812 | GTF2H4 |  |  | 13888 | 0.776 | 0.2939 | No |
| 423 | 103233294 | HSF4 |  |  | 13913 | 0.774 | 0.2939 | No |
| 424 | 103221830 | PPP1R10 |  |  | 13924 | 0.773 | 0.2945 | No |
| 425 | 103221504 | CCND3 |  |  | 14137 | 0.760 | 0.2859 | No |
| 426 | 103219526 | SMARCA2 |  |  | 14155 | 0.760 | 0.2863 | No |
| 427 | 103233592 | POLR2E |  |  | 14161 | 0.759 | 0.2871 | No |
| 428 | 103246092 | LOC103246092 |  |  | 14215 | 0.756 | 0.2858 | No |
| 429 | 103238679 | MDM1 |  |  | 14313 | 0.751 | 0.2824 | No |
| 430 | 103227932 | SETMAR |  |  | 14316 | 0.751 | 0.2834 | No |
| 431 | 103217163 | BAZ2B |  |  | 14320 | 0.751 | 0.2844 | No |
| 432 | 103223088 | TFIP11 |  |  | 14481 | 0.740 | 0.2781 | No |
| 433 | 103216413 | POLL |  |  | 14509 | 0.739 | 0.2779 | No |
| 434 | 103238749 | LOC103238749 |  |  | 14557 | 0.736 | 0.2769 | No |
| 435 | 103225264 | MED18 |  |  | 14582 | 0.734 | 0.2768 | No |
| 436 | 103226146 | POLM |  |  | 14598 | 0.733 | 0.2772 | No |
| 437 | 103224781 | TCEANC2 |  |  | 14602 | 0.733 | 0.2781 | No |
| 438 | 103220447 | GEMIN6 |  |  | 14608 | 0.732 | 0.2789 | No |
| 439 | 103243294 | HEXIM2 |  |  | 14622 | 0.731 | 0.2794 | No |
| 440 | 103244664 | IK |  |  | 14771 | 0.723 | 0.2737 | No |
| 441 | 103239270 | CUNH12orf43 |  |  | 14811 | 0.720 | 0.2729 | No |
| 442 | 103238936 | NR1H4 |  |  | 14828 | 0.719 | 0.2732 | No |
| 443 | 103217376 | HOXD9 |  |  | 14854 | 0.717 | 0.2731 | No |
| 444 | 103242808 | TBX2 |  |  | 15043 | 0.705 | 0.2655 | No |
| 445 | 103248782 | LOC103248782 |  |  | 15102 | 0.701 | 0.2639 | No |
| 446 | 103228065 | H1FOO |  |  | 15130 | 0.699 | 0.2637 | No |
| 447 | 103239669 | RXRA |  |  | 15149 | 0.698 | 0.2638 | No |
| 448 | 103233248 | ESRRA |  |  | 15193 | 0.695 | 0.2629 | No |
| 449 | 103237156 | CCNE2 |  |  | 15194 | 0.695 | 0.2639 | No |
| 450 | 103215858 | LOC103215858 |  |  | 15308 | 0.688 | 0.2597 | No |
| 451 | 103243081 | ADRM1 |  |  | 15331 | 0.687 | 0.2597 | No |
| 452 | 103242357 | TCEA2 |  |  | 15381 | 0.684 | 0.2584 | No |
| 453 | 103233492 | ZNF276 |  |  | 15470 | 0.677 | 0.2554 | No |
| 454 | 103227319 | NCAPG2 |  |  | 15475 | 0.676 | 0.2562 | No |
| 455 | 103221944 | H1-5 |  |  | 15563 | 0.671 | 0.2531 | No |
| 456 | 119625204 | LOC119625204 |  |  | 15584 | 0.669 | 0.2532 | No |
| 457 | 103227532 | SLX4 |  |  | 15610 | 0.668 | 0.2530 | No |
| 458 | 103246880 | MAFK |  |  | 15653 | 0.665 | 0.2521 | No |
| 459 | 103231614 | TCEANC |  |  | 15656 | 0.665 | 0.2529 | No |
| 460 | 103241276 | CDKN1C |  |  | 15685 | 0.663 | 0.2526 | No |
| 461 | 103236235 | NR1H3 |  |  | 15708 | 0.661 | 0.2525 | No |
| 462 | 103232725 | LOC103232725 |  |  | 15819 | 0.653 | 0.2484 | No |
| 463 | 103233679 | NFIC |  |  | 15857 | 0.650 | 0.2477 | No |
| 464 | 103243189 | LOC103243189 |  |  | 15880 | 0.648 | 0.2476 | No |
| 465 | 103230536 | TBX6 |  |  | 15925 | 0.645 | 0.2465 | No |
| 466 | 103222658 | GATA6 |  |  | 15927 | 0.645 | 0.2474 | No |
| 467 | 103233330 | SYCE1L |  |  | 15993 | 0.640 | 0.2454 | No |
| 468 | 103237565 | MAFA |  |  | 16047 | 0.636 | 0.2439 | No |
| 469 | 119620869 | HSFX3 |  |  | 16155 | 0.631 | 0.2399 | No |
| 470 | 103242195 | MED11 |  |  | 16268 | 0.623 | 0.2357 | No |
| 471 | 103224914 | TOE1 |  |  | 16318 | 0.619 | 0.2343 | No |
| 472 | 103231938 | SUV39H1 |  |  | 16324 | 0.619 | 0.2350 | No |
| 473 | 103238391 | ESPL1 |  |  | 16329 | 0.619 | 0.2357 | No |
| 474 | 103218393 | TEAD4 |  |  | 16335 | 0.618 | 0.2363 | No |
| 475 | 103217956 | LOC103217956 |  |  | 16416 | 0.612 | 0.2336 | No |
| 476 | 103215682 | LOC103215682 |  |  | 16678 | 0.593 | 0.2225 | No |
| 477 | 103248274 | SESN3 |  |  | 16767 | 0.586 | 0.2193 | No |
| 478 | 103234009 | NFIX |  |  | 16790 | 0.584 | 0.2191 | No |
| 479 | 103238213 | VDR |  |  | 16850 | 0.580 | 0.2173 | No |
| 480 | 103240827 | PNLDC1 |  |  | 16883 | 0.578 | 0.2166 | No |
| 481 | 103234183 | ELL |  |  | 16926 | 0.575 | 0.2155 | No |
| 482 | 103215868 | LOC103215868 |  |  | 16956 | 0.573 | 0.2150 | No |
| 483 | 103218031 | SP110 |  |  | 16960 | 0.573 | 0.2157 | No |
| 484 | 119626591 | LOC119626591 |  |  | 17021 | 0.567 | 0.2138 | No |
| 485 | 103238432 | NFE2 |  |  | 17029 | 0.567 | 0.2143 | No |
| 486 | 103244337 | LOC103244337 |  |  | 17047 | 0.567 | 0.2143 | No |
| 487 | 103230318 | ELF3 |  |  | 17069 | 0.566 | 0.2142 | No |
| 488 | 103218034 | LOC103218034 |  |  | 17136 | 0.563 | 0.2120 | No |
| 489 | 103245682 | LOC103245682 |  |  | 17240 | 0.555 | 0.2081 | No |
| 490 | 103241772 | RARB |  |  | 17261 | 0.553 | 0.2080 | No |
| 491 | 103232389 | LOC103232389 |  |  | 17277 | 0.551 | 0.2081 | No |
| 492 | 103245694 | LOC103245694 |  |  | 17387 | 0.542 | 0.2039 | No |
| 493 | 103218460 | POLD4 |  |  | 17407 | 0.540 | 0.2038 | No |
| 494 | 103245615 | LOC103245615 |  |  | 17570 | 0.527 | 0.1971 | No |
| 495 | 103220028 | INO80B |  |  | 17649 | 0.519 | 0.1943 | No |
| 496 | 103239112 | RAD9B |  |  | 17658 | 0.519 | 0.1947 | No |
| 497 | 103218667 | RAD9A |  |  | 17676 | 0.517 | 0.1946 | No |
| 498 | 103242473 | MED9 |  |  | 17702 | 0.515 | 0.1942 | No |
| 499 | 103225019 | CITED4 |  |  | 17880 | 0.500 | 0.1869 | No |
| 500 | 103223797 | LOC103223797 |  |  | 17964 | 0.492 | 0.1838 | No |
| 501 | 103234225 | PBX4 |  |  | 18055 | 0.485 | 0.1804 | No |
| 502 | 103237929 | MEIG1 |  |  | 18114 | 0.479 | 0.1784 | No |
| 503 | 103216261 | TBX10 |  |  | 18240 | 0.466 | 0.1733 | No |
| 504 | 103230642 | TBX19 |  |  | 18269 | 0.463 | 0.1727 | No |
| 505 | 103218115 | LOC103218115 |  |  | 18284 | 0.462 | 0.1728 | No |
| 506 | 119619376 | LOC119619376 |  |  | 18347 | 0.455 | 0.1706 | No |
| 507 | 103234789 | XRCC1 |  |  | 18452 | 0.445 | 0.1665 | No |
| 508 | 103231695 | CCNF |  |  | 18558 | 0.434 | 0.1623 | No |
| 509 | 103245959 | LOC103245959 |  |  | 18617 | 0.427 | 0.1602 | No |
| 510 | 103233348 | MAF |  |  | 18628 | 0.426 | 0.1604 | No |
| 511 | 103228304 | TNP2 |  |  | 18659 | 0.424 | 0.1596 | No |
| 512 | 103238423 | HOXC9 |  |  | 18695 | 0.423 | 0.1586 | No |
| 513 | 103222264 | FAM50B |  |  | 18880 | 0.404 | 0.1508 | No |
| 514 | 119621450 | LOC119621450 |  |  | 18884 | 0.403 | 0.1512 | No |
| 515 | 103234625 | CUNH19orf33 |  |  | 18902 | 0.402 | 0.1510 | No |
| 516 | 103232734 | LOC103232734 |  |  | 19011 | 0.392 | 0.1467 | No |
| 517 | 103246024 | MAFB |  |  | 19096 | 0.381 | 0.1434 | No |
| 518 | 119625820 | LOC119625820 |  |  | 19333 | 0.358 | 0.1331 | No |
| 519 | 103224140 | LOC103224140 |  |  | 19681 | 0.318 | 0.1177 | No |
| 520 | 103220197 | LOC103220197 |  |  | 19699 | 0.316 | 0.1174 | No |
| 521 | 103232186 | NAP1L2 |  |  | 19715 | 0.314 | 0.1171 | No |
| 522 | 119620729 | LOC119620729 |  |  | 19763 | 0.308 | 0.1154 | No |
| 523 | 119628108 | LOC119628108 |  |  | 19774 | 0.307 | 0.1154 | No |
| 524 | 103224018 | RORC |  |  | 19847 | 0.296 | 0.1125 | No |
| 525 | 103240732 | ESR1 |  |  | 19909 | 0.285 | 0.1102 | No |
| 526 | 103215870 | ADIRF |  |  | 20052 | 0.265 | 0.1040 | No |
| 527 | 103230290 | MYOG |  |  | 20159 | 0.248 | 0.0995 | No |
| 528 | 103225397 | TCEA3 |  |  | 20255 | 0.232 | 0.0955 | No |
| 529 | 103237881 | EHF |  |  | 20347 | 0.219 | 0.0917 | No |
| 530 | 103247498 | LOC103247498 |  |  | 20405 | 0.210 | 0.0894 | No |
| 531 | 103232396 | NXF3 |  |  | 20526 | 0.185 | 0.0842 | No |
| 532 | 103242329 | RNF227 |  |  | 20776 | 0.134 | 0.0730 | No |
| 533 | 103225320 | LOC103225320 |  |  | 20829 | 0.121 | 0.0707 | No |
| 534 | 119622904 | LOC119622904 |  |  | 20844 | 0.117 | 0.0703 | No |
| 535 | 103221630 | SPDEF |  |  | 20853 | 0.115 | 0.0701 | No |
| 536 | 103221590 | ETV7 |  |  | 20855 | 0.114 | 0.0702 | No |
| 537 | 103232253 | TBX22 |  |  | 21077 | 0.000 | 0.0601 | No |
| 538 | 103229373 | ESRRB |  |  | 21107 | 0.000 | 0.0588 | No |
| 539 | 103236685 | LOC103236685 |  |  | 21186 | 0.000 | 0.0552 | No |
| 540 | 103239475 | LOC103239475 |  |  | 21233 | 0.000 | 0.0531 | No |
| 541 | 103239887 | NR5A1 |  |  | 21275 | 0.000 | 0.0512 | No |
| 542 | 103247969 | LOC103247969 |  |  | 21507 | 0.000 | 0.0406 | No |
| 543 | 119620979 | LOC119620979 |  |  | 21639 | 0.000 | 0.0346 | No |
| 544 | 119625823 | LOC119625823 |  |  | 21746 | 0.000 | 0.0298 | No |
| 545 | 103239267 | LOC103239267 |  |  | 21998 | 0.000 | 0.0183 | No |
| 546 | 103234836 | CBLC |  |  | 22179 | 0.000 | 0.0101 | No |
| 547 | 119622650 | LOC119622650 |  |  | 22211 | 0.000 | 0.0086 | No |
| 548 | 119624495 | LOC119624495 |  |  | 22355 | 0.000 | 0.0021 | No |
| 549 | novel.532 | - |  |  | 22382 | 0.000 | 0.0009 | No |
Table: GSEA details
[plain text format]

  

Fig 2: NUCLEUS(GO:0005634)
  
Blue-Pink O' Gram in the Space of the Analyzed GeneSet

  

Fig 3: NUCLEUS(GO:0005634): Random ES distribution
  
Gene set null distribution of ES for
 **NUCLEUS(GO:0005634)**
